# Patient‐derived preclinical models to develop immunotherapies

**DOI:** 10.1002/1878-0261.13470

**Published:** 2023-06-15

**Authors:** Joan Seoane

**Affiliations:** ^1^ Vall d'Hebron Institute of Oncology (VHIO), Vall d'Hebron University Hospital, Universitat Autònoma de Barcelona, ICREA, CIBERONC Spain

**Keywords:** immuno‐oncology, immunotherapy, patient‐derived models, preclinical models, translational models, tumor microenvironment

## Abstract

Cancer immunotherapy has revolutionized the treatment of some malignancies. Yet, many tumors do not unfortunately respond to immune‐based therapies. Deeper insights into the biology of the immune response to cancer are required to identify novel therapeutic targets and advance immuno‐oncology. To do so, we need to study cancer in patient‐derived models that can faithfully recapitulate and capture the complexity and heterogeneity of the tumor immune ecosystem. Platforms facilitating the analysis of the human tumor immune microenvironment of individual patients are crucial. Patient‐derived models are fundamental not only to better understand the biology of the cancer immune system but also to discern the mechanism of action of therapeutic compounds and perform preclinical studies toward improving the success of subsequent clinical testing. In this viewpoint, I present a brief review of patient‐derived models for cancer immunotherapy.

## Graphical abstract

Deeper insights into the biology of the immune response to cancer are required to identify novel therapeutic targets and advance immuno‐oncology. To do so, we need to study cancer in patient‐derived models that can faithfully recapitulate and capture the complexity and heterogeneity of the tumor immune ecosystem. Platforms facilitating the analysis of the human tumor immune microenvironment of individual patients are crucial. In this viewpoint, I present a brief review of patient‐derived models for cancer immunotherapy.

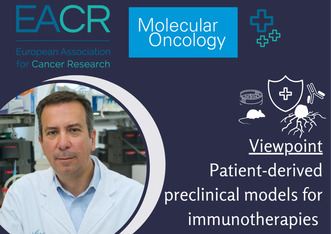

**Abbreviations**
GEMM: genetically engineered mouse model
IL6: interleukin 6
NSG: NOD SCID gamma
PDTTC: patient‐derived tumor tissue culture
PDX: patient‐derived xenograft

Immunotherapies induce robust and durable antitumor activity in a subset of cancer patients; however, the majority of patients remain refractory to treatment and may require novel strategies that sensitize tumors to immune‐based interventions [1].

The capacity of the immune system to mount an effective antitumor response is largely determined by a complex and poorly understood interplay of various factors, including the presence and activation status of immune cells, the local cytokine milieu, and the dynamic tumor–host interactions in the tumor microenvironment [2, 3]. Within this intricate ecosystem, the molecular and cellular mechanisms that induce immune suppression vary depending on tumor type and can evolve considerably over time, representing a challenging area of research. Achieving deeper insights into the molecular mechanisms of tumor and immune system interaction is fundamental to the design and the development of more effective immunotherapies and extending the promise of immuno‐oncology to an increasing number of patients across tumor types.

Seminal studies exploring the immune contexture and mechanism characteristic of localized, primary tumors have produced a large body of knowledge on tumor–immune interactions promoting tumor growth and invasion in the local environment [3]. These insights have paved the way for the development of therapies that are effective in a relatively small patient population. Much more research centered on the cancer immune system is still required to discern the molecular mechanisms implicated in tumor immune evasion. These efforts will spur the identification of novel therapeutic targets, leading to the development of new therapies, and help to expand the number of patients who could benefit from immuno‐oncology.

Research on the immune response to cancer has been mainly hindered by the lack of direct analysis of human samples and access to physiologically‐relevant models that fully recapitulate the tumor microenvironment of patients. The characterization of human samples is crucial to study and understand the reality of cancer immune evasion. Importantly, the unique microenvironment of human tumors, including the diverse and specific cellular composition and immune components, makes the modeling of the cancer immune system a challenging task.

In the study of the immune tumor microenvironment, many current preclinical models have important limitations. Cell line xenograft models in immunocompromised mice cannot be used for these studies. Even immunocompetent mouse allograft tumor models and spontaneous cancer models in genetically engineered mouse models (GEMM) do not mimic the characteristics of the immune tumor microenvironment, and their use entails significant limitations.

Tumors generated from syngeneic tumor cell lines and in GEMMs do not recapitulate the complexity and heterogeneity of human cancer. They exhibit very limited intratumor heterogeneity (a critical factor in the response to the immune system) and do not capture the adaptation of the tumor to the immune system throughout the long history of tumor development in human cancer. Human tumors take many years to develop, and they adapt to and are edited by the immune system. By contrast, both allograft and GEMM models do not undergo this type of long coevolution that determines the intricate immune ecosystem present in human tumors. Moreover, the mouse immune compartments differ from those of humans, and some cytokines do not signal across species (i.e., IL6) [4]. All these factors greatly preclude the right interpretation of results observed in these models.

Importantly, allograft models and GEMMs are used in the preclinical evaluation of pharmacological compounds. Their limitations can lead to research findings that cannot be easily translated into the clinic when novel therapies are tested in clinical trials. The understanding of the mechanism of action of therapeutic compounds is fundamental for the successful development of clinical trials and requires optimal preclinical models.

With the aim of getting closer to the reality of human cancer, several patient‐derived functional models have been developed: tumor organoids or tumoroids [5, 6], patient‐derived tumor tissue cultures (PDTTCs) [7, 8], patient‐derived xenografts (PDX) [9, 10, 11], plus leukocytes and humanized PDX [12, 13, 14] (Fig. 1). These models are generated thanks to multidisciplinary and largely hospital‐based teams including surgeons, oncologists, pathologists, and translational researchers; and they can facilitate the study of the immune response to cancer. However, each of the models exhibit their own limitations and only their combined use will achieve a better understanding of the complex immune ecosystem.

**Fig. 1:**
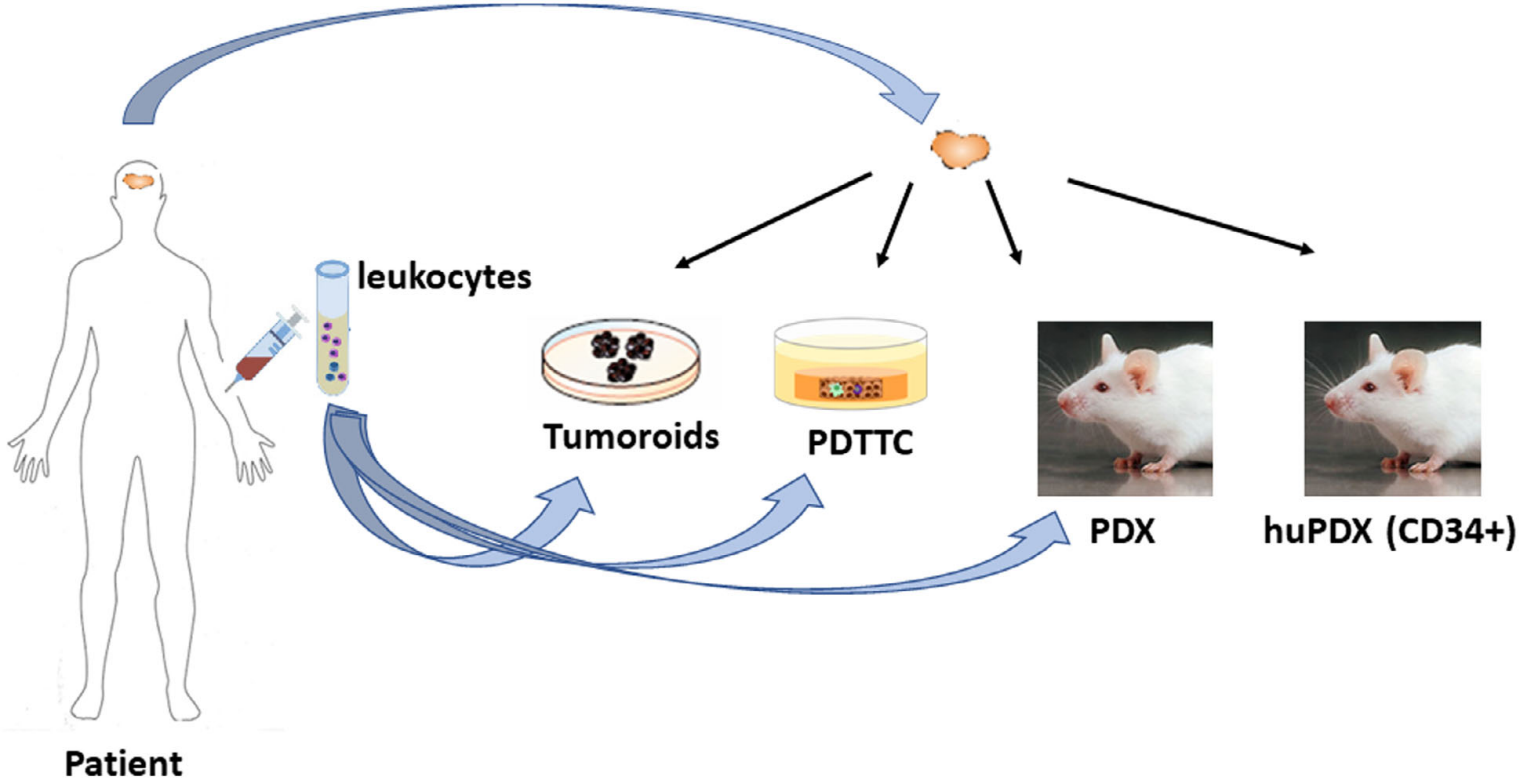
Scheme of patient‐derived models.

## 1 Tumoroids

Tumor cells from surgical samples are cultured in a special medium in the absence of serum. Tridimensional multicellular suspension spheres can be generated. These cultures can be passaged, frozen, and amplified for future experiments. They can be implanted in mice to generate tumors and can be infected with lentivirus expressing luciferase to monitor tumor growth. These cultures can be cocultured with leukocytes from the same patient that donated the tumor and the immune cell–tumor cell interactions can be analyzed. While these models recapitulate the complexity and heterogeneity of human cancer cells, they lack the tumor microenvironment, which can be crucial to the immune response to cancer.

## 2 PDTTCs

A fraction of the tumor is cut with a vibratom into 350‐μm‐thick slices and put into semidry culture conditions. These organotypic models allow the short‐term culture (7–10 days) of sections of tumors and maintain the tissue architecture and the stroma (infiltrating immune cells and endothelial cells among others) from the tumor of the patient. These models can be cocultured with autologous leukocytes, and the immune cell–tumor cell interactions can be analyzed. The viability of PDTTCs should be thoroughly monitored and should be higher than 80% for the experiment to proceed. These are therefore optimal models for the study of the tumor microenvironment in the context of the actual tumor of the patient, yet they lack the physiological aspects of *in vivo* models.

## 3 PDX plus leukocytes

Patient‐derived xenografts models are generated through the subcutaneous or orthotopic inoculation of small fresh tumor fragments in immune‐compromised mice (i.e., NOD SCID gamma (NSG) mice). The main limitation of this model is that NSG mice lack an immune system. In order to circumvent this problem, leukocytes can be implanted in mice (optimally from the same patient that donated the tumor). Although these procedures are known to cause graft‐versus‐host disease beginning 2–5 weeks after injection, short‐term experiments can be performed to address specific questions.

## 4 Humanized PDX

Patient‐derived xenografts models can be generated in CD34^+^ humanized mice. These humanized mouse models are generated first through the eradication of the mouse immune system to then reconstitute the human immune system by inoculating CD34^+^ hematopoietic stem cells. Yet, in this case, the immune cell reconstitution is not fully completed and, for example, the human myeloid compartment is not well re‐established. Moreover, the model does not present with the patient's autologous immune cells, and this limits the interpretation of the immune cell–tumor cell interactions. Moreover, the long coevolution of the tumor immune microenvironment that takes place in the human tumor is also missing.

All these patient‐derived models have limitations but are complementary and when used in combination can allow the comprehensive study of the immune tumor microenvironment. In addition, each model should be used based on the question that is being addressed. Specific questions will require specific patient‐derived models or combined approaches to circumvent the limitations of each model and leverage complementary strengths.

These models can be used for the preclinical testing of novel therapeutic compounds. Nowadays, many pharmaceutical companies are not usually using patient‐derived models. This challenges the translation of preclinical results into clinical studies, and without fully elucidating the mechanism of action of a compound, many trials may fail. The use of the right model or combination of patient‐derived models can advance insights into the biology of the immune response to cancer and help make clinical trials more successful.

## Conflict of interest

JS is a cofounder of Mosaic Biomedicals and has ownership interests from Mosaic Biomedicals and Northern Biologics. JS received grant/research support from Mosaic Biomedicals, Northern Biologics, Roche/Glycart, Hoffmann la Roche, and AstraZeneca.
